# HIV Nef Expression Down-modulated GFAP Expression and Altered Glutamate Uptake and Release and Proliferation in Astrocytes

**DOI:** 10.14336/AD.2022.0712

**Published:** 2023-02-01

**Authors:** Kelly M. Wilson, Johnny J. He

**Affiliations:** Department of Microbiology and Immunology, Center for Cancer Cell Biology, Immunology and Infection, School of Graduate and Postdoctoral Studies, Rosalind Franklin University, Chicago Medical School, North Chicago, IL 60064, USA

**Keywords:** HIV Nef, GFAP, transcription control, glutamate uptake and release, proliferation, astrocytes

## Abstract

HIV infection of astrocytes leads to restricted gene expression and replication but abundant expression of HIV early genes Tat, Nef and Rev. A great deal of neuroHIV research has so far been focused on Tat protein, its effects on astrocytes, and its roles in neuroHIV. In the current study, we aimed to determine effects of Nef expression on astrocytes and their function. Using transfection or infection of VSVG-pseudotyped HIV viruses, we showed that Nef expression down-modulated glial fibrillary acidic protein (GFAP) expression. We then showed that Nef expression also led to decreased GFAP mRNA expression. The transcriptional regulation was further confirmed using a GFAP promoter-driven reporter gene assay. We performed transcription factor profiling array to compare the expression of transcription factors between Nef-intact and Nef-deficient HIV-infected cells and identified eight transcription factors with expression changes of 1.5-fold or higher: three up-regulated by Nef (Stat1, Stat5, and TFIID), and five down-regulated by Nef (AR, GAS/ISRE, HIF, Sp1, and p53). We then demonstrated that removal of the Sp1 binding sites from the GFAP promoter resulted in a much lower level of the promoter activity and reversal of Nef effects on the GFAP promoter, confirming important roles of Sp1 in the GFAP promoter activity and for Nef-induced GFAP expression. Lastly, we showed that Nef expression led to increased glutamate uptake and decreased glutamate release by astrocytes and increased astrocyte proliferation. Taken together, these results indicate that Nef leads to down-modulation of GFAP expression and alteration of glutamate metabolism in astrocytes, and astrocyte proliferation and could be an important contributor to neuroHIV.

Over 37 million people worldwide are infected with HIV [1]. Introduction of combination antiretroviral therapy has extended the lifespan of HIV-infected individuals, leading to increased incidents of minor cognitive and motor dysfunction [2-7]. More than 50% of HIV-infected individuals exhibit some form of neurological symptom collectively known as neuroHIV [8-10]. Hallmarks observed in neuroHIV include impairments in neurobehaviors (motor ability and memory) and neuropathology such as neuroinflammation, impaired neuronal integrity, and astrocytosis [4, 5, 11].

Astrocytes are the most abundant cell of the central nervous system with two primary functions. Firstly, they constitute a main component of the blood brain barrier providing structure and support. This physical network provides a scaffold for molecular trafficking between cells throughout the entire brain [12-14]. Secondly, astrocytes serve to maintain homeostatic balance of molecules and ions, such as ATP, calcium and glutamate within and around the tripartite of neuronal synaptic clefts and astrocyte [15-17]. Monitoring and regulating this are critically important for proper neuron function and overall brain health. Only recently have studies begun diving into how the major astrocytic protein, glia fibrillary acidic protein (GFAP), not only serves as a cellular marker of astrocytes, but also plays a major role in regulating astrocyte functionality [18-24]. One such example is that post- translational modification hyperpalmitoylation of GFAP leads to increased GFAP expression, which is linked to astrocyte proliferation, and neurodegeneration [25].

HIV infection of astrocytes is generally thought to be restricted with little production of infectious progeny viruses, but with abundant expression of HIV early genes such as Nef [26-30]. Nef is a 27 kDa myristolated protein[26, 31]. It is indispensable for HIV pathogenesis through regulation of expression of immune molecules such as CD4 and MHC I, viral infectivity, and intracellular signaling [32-34]. It is also linked to the compromised integrity of the blood brain barrier (BBB), increased expression of monocyte chemoattractant protein-1 (MCP-1) in astrocytes, and the increased sensitivity of astrocytes to oxygen species [30, 35-40]. Nef expression alone in mice leads to an AIDS-like disease; while Nef deletion or defect is linked to lower viral load and attenuated diseases in humanized mice, non-human primates and humans [41-44]. However, the roles of Nef in neuroHIV and the underlying mechanisms are not entirely clear.

HIV infection of astrocytes and resulting functional impairments during neuroHIV have been proposed to contribute to the disease progression through a myriad of molecular mechanisms. In this study, we aimed to understand if Nef expression would alter GFAP expression and astrocyte function. By introducing Nef into astrocytes via transfection or HIV infection, we demonstrated that Nef expression down-modulated GFAP expression through regulation of transcription factor Sp1 and altered glutamate uptake in astrocytes. These findings indicate that abundant Nef expression in astrocytes resulting from HIV infection plays important roles in neuroHIV pathogenesis and may provide new evidence to further support astrocyte dysfunction as a major mechanism of neuroHIV as well as GFAP expression as a functional marker of astrocytes.

## MATERIALS AND METHODS

### Cells and transfections

Human embryonic kidney cell line 293T and astrocytic cell lines U373 and SVGA were purchased from American Tissue Culture Collection (Manassas, VA) and maintained at 37°C with 5% CO2 in Dulbecco's Modified Eagle's Medium (Corning Inc., Corning, NY) supplemented with 1% penicillin/streptomycin (Sigma-Aldrich, Burlington, MA) and 10% fetal bovine serum (R&D Systems, Minneapolis, MN). Mouse primary astrocytes were collected from the brain tissues of prenatal day 18-20 wild-type mouse fetus, cultured in F12K media (Corning) for two passages, and used. Human fetal primary astrocytes (HPA) were isolated from aborted human fetal brain tissues provided by the Laboratory of Developmental Biology, supported by the NIH Award Number 5R24HD000836 from the Eunice Kennedy Shriver National Institute of Child Health and Human Development. 293T and SVGA were transfected by the standard calcium phosphate precipitation transfection method and estimated to have a transfection efficiency of >95% and ~75%, respectively. The calcium phosphate transfection method for U373 and HPA cells resulted in low efficiency of gene expression (~20% and ~5%). To circumvent this, U373 and HPA were transfected by Lipofectamine 3000 transfection reagents (Cat. # L3000015, ThermoFisher Scientific, Waltham, MA) and media was replaced 16 hr post transfection the cells were cultured for 24-48 hr with an estimated >95% and ~80% transfection efficiency. For both calcium phosphate and lipofectamine 3000 methods, a total of 2.5ug DNA was used and transfection efficiency was estimated by co-transfection of pC3.GFP plasmid and counting the GFP+ cells under a fluorescence microscope.

### Plasmids

pcDNA3 (pC3), pc3.GFP, and pNef.Myc were previously described[45]. HIV reporter virus vectors GAGi and NLGi were generous gifts from Dr. B. K. Chen of Mount Sinai School of Medicine [34, 46]. These two HIV reporter vectors were derived from the pNL4-3 HIV vector. To construct GAGi, the green flourescent protein (GFP) reporter gene was inserted within the Gag gene between matrix and caspid allowing for GFP to be directly packaged into virions for direct monitoring of the infection efficiency [28, 47, 48]. For NLGi, GFP was inserted immediately preceeding the open reading frame of the Nef gene, allowing GFP to be expressed as an early gene [28, 47]. To create the Nef deficient counter parts, GAGi.Nef- and NLGi.Nef-, GAGi and NLGi were subjected to Xho1 digestion, and filled-in with T4 ligase, efficiently preventing expression of Nef [28, 47]. Murine GFAP promoter driven luciferase plasmid (pGFAP.luc) was a generous gift from Dr. Michael Brenner of University of Alabama at Birmingham [49]. All three Sp1 DNA binding sites were deleted from the pGFAP.luc plasmid to create the Sp1-deleted reporter plasmid pΔSp1-GFAP.luc using a Q5 Site-Directed Mutagenesis kit (New England Biolabs, Ipswich, MA) and primers 5’- TTC CCT TCG ATG CTT TCC GAG-3’ and 5’-TTC TGA CCA TTA TGT CTA TGC C-3’.

### Production of pseudotyped HIV reporter viruses and infection

293T were transfected with pVSVg and GAGi or pNef-deficient GAGi (GAGi.Nef-) at a 1:7 ratio using the standard calcium phosphate precipitation method. The transfection medium was replaced with fresh medium 16 hr post transfection, the cells were cultured for 2 days, and the culture medium was collected. After removal of cell debris by a brief centrifugation, the supernatants were aliquoted and stored at -80°C as virus stocks. Pseudotyped NLGi and NLGi.Nef- were similarly prepared. The reverse transcription activity assay (RT assay) was performed using culture supernatant for virus quantitation. Briefly, virus was pelleted from 1 ml supernatants by centrifugation at 14,000 *g*, 4°C for 1 hr, suspended in 10 μl dissociation buffer (1 mm DTT, 0.25 M KCL, 50 nM Tris.HCl, pH7.5, 0.25% Triton-X100, 20% Glycerol) and subjected to 5 cycles of freeze/thaw. Then, 40 μl RT reaction buffer (7.2mM Tris.HCl, pH 7.5, 0.00075% Triton-X100, 11.26 mM MgCl_2_ containing polyadenylic acid x pentadecathymidylic acid (Poly A, Cat. # 10108677001, Roche Diagnostics; Barrington, IL) and deoxythymidine 5'-triphosphate, tetrasodium salt (Cat. # NET221X005MC, Perkin Elmer; Naperville, IL) was added, and the mixture was incubated at 37°C for 1 hr and spotted onto DE81 ion exchange chromatography paper, followed by sequential rinses in 2X saline-sodium citrate buffer and 100% ethanol and air drying. The ion exchange paper was added to a 1.5 ml microcentrifuge tube along with the scintillation counting fluid (Microscint PS, Cat. # 6013631, Perkin Elmer; Naperville, IL) and counted for the tridium activity using a scintillation counter (Microbeta2, Cat # 2450 Microplate Counter, Perkin Elmer, Naperville, IL). The RT activity expression was recorded as counts per minute (CPM) per ml. For infection, SVGA, U373, or human primary fetal astrocytes were seeded at a density to reach 80-85% confluency following culturing for 24 hr. Medium was changed and 18,000 (for a 24-well plate) or 80,000 (for a 6-well plate) CPM RT equivalent pseudotyped virus was added. Medium was changed after 16 hr and cells incubated for an additional 24-48 hr.

### Western blotting

Transfected or infected cells in 6-well plate were rinsed twice with ice-cold 1X phosphate buffer saline (PBS), RIPA buffer (20 mM Tris.HCl, pH 8.0, 150 mM NaCl, 1 mM EDTA, 1 mM EGTA, 1% Triton-X100) was added and cells incubated on ice for 10 min. Then, the cells were scraped off and collected in a 1.5 m microcentrifuge tubes. The mixture was incubated on ice for additional 10 min and centrifuged at 10,000 *g*, 4°C for 10 min. The supernatants were collected and saved as cell lysates for Western blotting. The protein concentration of the cell lysates was determined using a Bradford chemi-luminescent assay kit (Bio-Rad, Hercules, CA) and absorbance determined at wavelength 595 on the iMark spectrophotometer (Bio-Rad; Hercules, CA). The cell lysates were separated on 8-12% SDS polyacrylamide gels and transferred to polyvinylidene fluoride membrane. The membrane was blocked in 5% non-fat milk buffer, first incubated in appropriate primary antibody at 4°C overnight and then in appropriate HRP-conjugated secondary antibody at room temperature for 4 hr, developed with Enhanced Chemiluminescence Reagents (Cat. # 32106, ThermoFisher Scientific), and visualized with a Bio-Rad Chemidoc MP imaging system (Bio-Rad). Primary antibodies used in the study were GFAP (anti-rabbit, 1:5000, Cat. #7260, Abcam, Waltham, MA), Nef (anti-mouse, 1:500, Cat. #1539; anti-rabbit, 1:500, Cat. #2949, NIH AIDS Reagents Program), p24 (anti-mouse,1:1000, NIH AIDS Reagents Program), and Sp1 (anti-rabbit, 1:200, Cat. # PA1-30332, Invitrogen, Waltham, MA), β-actin (anti-mouse, 1:2000, Cat. #A1978, Sigma Aldrich) and GAPDH (anti-mouse, 1:2000, Cat. # 6C5, Santa Cruz, Dallas, TX). Secondary HRP-conjugated antibodies were anti-mouse (1:2000, Cat. # NA931, Millipore Sigma, Burlington, MA) or anti-rabbit (1:2000, Cat.# 4050-05, Southern Biotech, Birmingham, AL).

### RNA isolation and qRT-PCR

Total RNA was isolated from transfected or infected cells using TRIzol Reagent (Cat. # 15596018, Invitrogen) according to the manufacturer’s instructions. cDNA was prepared with 1 μg RNA in 20 μl reaction using an iScript cDNA synthesis kit (Cat. # 1708890, Bio-Rad). Real-time PCR was performed using gene-specific primers and the SsoAdvanced Universal SYBR Green qPCR Super Mix (Cat. # 1725270, Bio-Rad). Fold changes in cycle threshold (∆∆Ct) values were calculated using the Ct values of the internal control GAPDH. Gene-specific qPCR primers were mouse GFAP 5’- TCT AAG TTT GCA GAC CTC ACA GA and 5’-ACT CCA GAT CGC AGG TCA A-3’, mouse GAPDH 5’-CAT GGC CTT CCG TGT TCC TA-3’ and 5’-CAT GGC CTT CCG TGT TCC TA-3’, human GFAP 5’-GCT TTG CCA GCT ACA TCG AG-3’ and 5’-GGT AGA CGT CTG CCA GCT TG-3’ and human GAPDH 5’-GAA ACT GTG GCG TGA TGG C-3’ and 5’- CCA GTG AGC TTC CCG TTC AG-3’.

### Luciferase reporter gene assay

U373 were plated at a density of 2.5 X10^5^ in a 12-well plate, transfected with pGFAP.luc or pΔSp1-GFAP.luc vectors using Lipofectamine 3000, cultured for 6 hr, and then replaced with fresh media. The cells were then infected with 60,000 cpm RT equivalent pseudotyped viruses in 600 μl by spinoculation at 805 x *g* for 1 hr, cultured at 37°C, 5% CO_2_ for 1 hr, and replaced with fresh media. The cells continued to culture for 48 hr, rinsed in ice-cold PBS, and then lysis buffer added (Cat. # E4030, Promega, Madison, WI). The cells were incubated at 4°C for 10 min, scraped off, collected in a 1.5 ml centrifuge tube, and continued to incubate on ice for 10 min. The mixture was briefly spun, the supernatants were collected as the cell lysates. The cell lysates were mixed with Firefly luciferase substrate (Cat. # E1500, Promega, Madison, WI) at a ratio of 5:1 and measured for the luciferase reporter gene activity using a luminometer (MGM Instrument, Hamden, CT). The luciferase activity was and normalized to the protein concentration and expressed as relative luminescence units (RLU/protein).

### Transcription factor profiling array

A two-part buffer system was used to obtain cytoplasmic extract with CE buffer (20mM HEPES, pH 7.8, 120 mM KCl, 2mM EDTA, 0.15% NP-40, 20% glycerol, 2mM DTT, 1x Pierce proteinase inhibitors (Cat. # A32963, Thermo Scientific, Waltham, Massachusetts) and nuclear extract with NE buffer (20 mM Tris.HCl, pH 8.0, 400 mM NaCl, 20% glycerol, 1% Triton-X100, 1 mM DTT, 1x Pierce proteinase inhibitors) from U373 that were infected with pseudotyped GAGi or GAGi.Nef-. Briefly, in a 6-well plate, media was removed, and cells were rinsed twice with ice-cold 1X phosphate buffer saline (PBS) before adding CE buffer and incubating on ice for 15 min at 4°C. Then, the cells were scraped off, collected in a 1.5 mL microcentrifuge tubes and incubated on ice for additional 10 min while shaking and then centrifuged at 10,000 x *g, at* 4°C for 10 min. This cytoplasmic lysate was removed and stored, and NE buffer was added to the remaining pellet, which was then subjected to sonication for 3 seconds, incubated on ice for 10 min, then centrifuged 10,000 x *g, at* 4°C for 10 min. Nuclear extract was used for transcription factor profiling array (Cat. # FA-1001, Signosis, Santa Clare, CA) according to the manufacturer’s instructions. Briefly, after obtaining protein concentration via BCA, 15 μg nuclear extract from GAGi- or GAGi.Nef--infected U373 was incubated with biotin-labeled DNA probes. The captured probes were then hybridized to a 96-well plate where each well was pre-coated with a single transcription factor complementary sequence, incubated with HRP-conjugated streptavidin, and quantitated for the amount of bound DNA probe using a 96-well plate reader (Bio-Tek Instruments, Winooski, VT). The luminescence reading (RLU) was normalized to Histone 3 levels determined from Western blotting of the same nuclear lysates. Two wells without DNA probe mixture were included as the background controls.

### Glutamate Uptake Assay

For infection, U373 or primary human fetal astrocytes were plated in a 24-well plate at a density of 0.75 X10^5^ per well, cultured for 24 hr (to reach 85% confluency) and infected with 18,000 CPM RT equivalent pseudotyped GAGi or GAGi.Nef- for 16 hr, then medium were replaced. The cells continued to culture for 24 hr. For transfection, U373 and human primary astrocytes were plated in a 24-well plate at a density of 0.9 x 10^5^ per well, cultured for 24 hr, and transfected with pC3/pc3.GFP or Nef/pc3.GFP, medium was replaced after 16 hr, and cells were cultured for an additional 24 hr. Medium was removed and cells washed twice with pre-warmed PBS before equilibrating cells at 37°C in Kreb’s bicarbonate buffer (10 mM glucose, 26.75 mM NaHCO_3_, 1.2 mM CaCl_2_, 124 mM NaCl, 4.6 mM KCl, 1.3 mM MgCl_2_.6H_2_O, 0.416 mM KH_2_PO_4_.2H_2_O, pH 7.4) for 30 min. Cells were then pulsed with a glutamate mix in Kreb’s buffer, containing ^3^H-Glutamate (1.4 mCi/ml, Cat # Net490250 µci-Perkin Elmer; Shelton, CT) and 0.5 µM unlabeled glutamate (Cat. # G2128, Sigma-Aldrich) for 20 min at 37°C. To stop the reaction and remove excess glutamate, cells were thoroughly washed with ice-cold PBS 5 times before lysing with 200 µl 0.1 N NaOH. Separate aliquots were taken for scintillation counting in duplicate and for protein concentration used for normalization. Intracellular ^3^H-glutamate was calculated as CPM/mg protein.

### Glutamate release assay

Cells were infected or transfected as described above for the glutamate uptake assay. Twenty-four hours after changing the media, 100 μl supernatant was collected. The supernatant was briefly centrifuged at 300 x *g* to remove unattached cells. To determine glutamate concentrations in the supernatant, an absorbance-based glutamate assay (Abcam, Cat. # 83389) was carried out according to the manufacturer’s protocol. Briefly, 20 μl supernatant was added to each well of a 96-well plate, followed by addition of 35 μl reaction mix (30 μl buffer, 4 μl developer, and 1 μl enzyme), gently mixed, and incubated at 37°C for 30 mins. The absorbance was taken at a wavelength of 450 nm. Concentrations were determined by extrapolating from the standard curve. To calculate the glutamate release, experimental samples concentrations were subtracted from the background control sample (media only). The glutamate concentration was normalized to the protein concentration of the corresponding whole cell lysates.

### MTT Assay

For infection, U373 were plated in a 48-well plate at a density of 0.38 x 10^5^ per well, cultured for 24 hr (to reach 85% confluency) and infected with 9,000 CPM RT equivalent VSVG-pseudotyped GAGi or GAGi.Nef- for 16 hr, then medium were replaced. The cells continued to culture for 24 hr. For transfection, U373 and HPA were plated in a 48-well plate at a density of 0.45 x 10^5^ per well, cultured for 24 hr, and transfected with pC3/pc3.GFP or Nef/pc3.GFP, medium was replaced after 16 hr, and cells were cultured for an additional 24 hr. Medium was replaced with full DMEM medium containing MTT reagent (1 mg/ml) and incubated at 37°C for 4 hr, at which time the reaction mixture was aspirated. Acid isopropanol (0.02 M HCl diluted in Isopropanol) was added to cells directly in the well, and allowed to fully dissolve by shaking at RT for 20 min. The plate was briefly centrifuged, and supernatant was transferred to a new 96-well plate in triplicate for absorbance reading at wavelength 595 nm, with the background substraction at 655 nm using an iMark plate reader (Bio-Rad).

**Figure 1.**
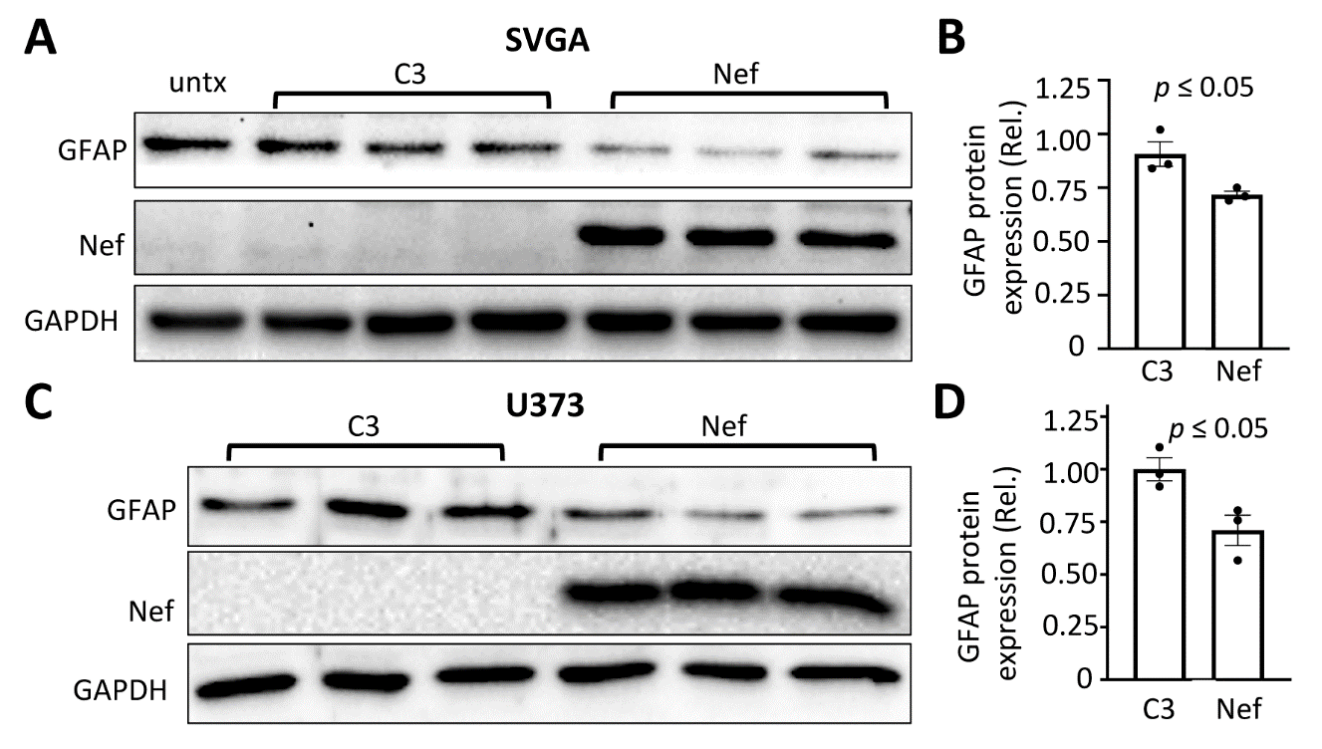
Effects of Nef expression on GFAP protein expression in human astrocytes. Human astrocytic cell lines SVGA (A) or U373 (C) were plated in a 6-well plate at a density of 2.5 x 10^5^ cells/well for SVGA or 3.0 x 10^5^ cells/well for U373, transfected with 2.5 μg pcDNA3 plasmid (C3) or pNef.Myc plasmid (Nef). The cell lysates were analyzed by Western blotting (A & C) against an anti-GFAP, Nef, GAPDH, or β-actin antibody. GFAP expression was quantitated, normalized to GAPDH or β-actin, and expressed as the relative values to the control (rel) (B & D). untx: untransfected cells. The data were Mean ± SEM and representative of three independent experiments (n=3).

### Data analysis

The software GraphPad Prism 9 was used to conduct statistical analysis. Unpaired two-tailed Student’s *t*-test (parametric) was performed for all two-way comparisons with normal distribution and equal variances among the groups. All values expressed as a Mean +/- SEM and were representative of three biological replicates (n=3). *p* value less than 0.05 was considered significant.

## RESULTS

### Nef Expression down-modulated GFAP expression in astrocytes

GFAP expression, the primary cellular marker for astrocytes, flutuates during disease states [18, 30, 31, 50]. Because of this and GFAP’s specificity to astrocytes, GFAP expression is commonly used as an indicator to monitor potential changes in astrocyte function. NeuroHIV induces changes in astrocytes, and HIV Nef is involved in this process [51, 52]. To understand whether Nef expression has direct effects on GFAP expression, we introduced Nef into astrocytes via transfection and determined the GFAP protein level 24 hr post media change by Western blotting. Nef expression in human astrocytic cell line SVGA led to a lower level of GFAP expression (~1.25 fold) compared to pcDNA (C3) transfected cells. GFAP expression was comparable between cell lysates of untransfected cells and C3 transfected cells demostrating that the transfection process did not contribute to the observed changes in GFAP expression in the presence of Nef (Fig. 1A & B). Similar results were obtained in another Nef-transfected human astrocyoma cell line U373 (Fig. 1C & D).

**Figure 2.**
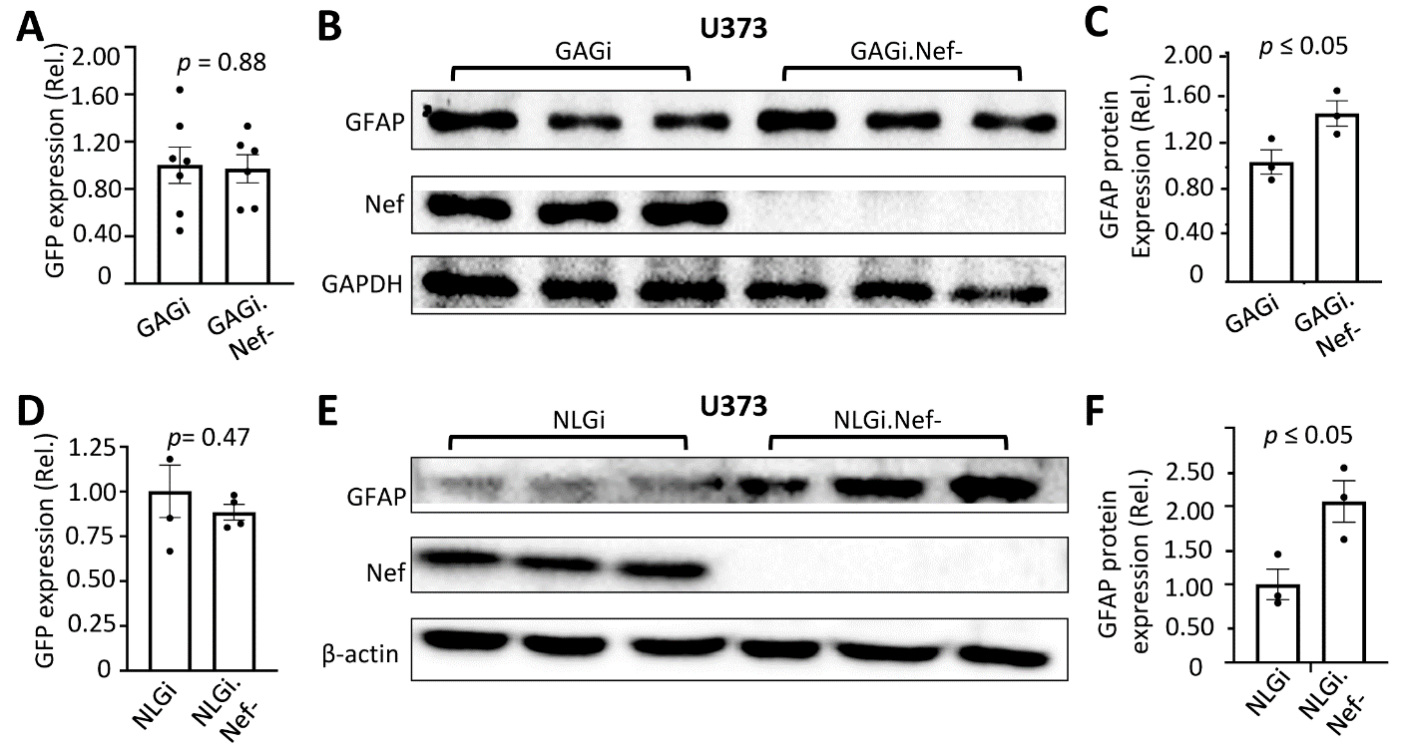
Effects of Nef expression on GFAP expression in human astrocytes in the context of HIV infection. U373 were infected with VSVG/GAGi and GAGi.Nef- (A-C) or NLGi or NLGi.Nef- (D-F). The cells were cultured for 48 hr, the GFP+ cells were estimated under a fluorescence microscope and expressed as a ratio to the total number of cells (A & D); or the cells were harvested for cell lysates and Western blotting against an anti-GFAP, Nef, GAPDH, or β-actin antibody (B & E). GFAP expression was quantitated, normalized to GAPDH or β-actin, and expressed as the relative values to the control (rel.) (C & F). The data were Mean ± SEM and representative of three independent experiments (n=3).

### Nef down-modulated GFAP expression in astrocytes in the context of HIV infection

**Figure 3.**
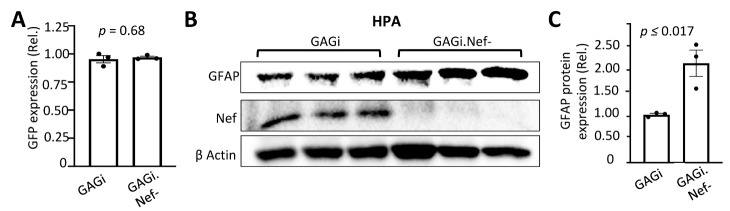
Effects of Nef on GFAP protein expression in human primary astrocytes in the context of HIV infection. Human primary fetal astrocytes (HPA) were infected with VSVG/GAGi and GAGi.Nef-. The cells were cultured for 48 hr, the GFP+ cells were estimated under a fluorescence microscope and expressed as a ratio to the total number of cells (A); or the cells were harvested for cell lysates and Western blotting against an anti-GFAP, Nef, or β-actin antibody (B). GFAP expression was quantitated, normalized to β-actin, and expressed as the relative values to the control (rel.) (C). The data were Mean ± SEM and representative of three independent experiments (n=3).

To determine the effects of Nef expression in the context of HIV infection, we infected U373 with VSVG-pseudotyped GAGi or GAGi.Nef- HIV reporter viruses in which the GFP reporter gene was inserted between MA and CA to allow for monitoring of infection efficiency [28, 47, 48]. Use of VSVG pseudotyping not only allowed a comparable high level of HIV infection of astrocytes (Fig. 2A), but also eliminated any differences of infectivity resulting from Serinc5/3 interaction with Nef and with HIV envelope gp120 [53-57]. Compared to VSVG/GAGi infection, VSV-G/GAGi.Nef- infection showed a higher level of GFAP protein expression (~1.4 fold) (Fig. 2B & C). To confirm this finding, U373 were infected with another VSVG-pseduotyped HIV reporter virus NLGi or its Nef-deficient counterpart NLGi.Nef-, in which GFP was inserted immediately upstream of the open reading frame of Nef gene allowing for GFP to expressed in the same pattern as Nef [28, 47]. There was a comparable infection efficiency between VSVG-pseudotyped NLGi and NLGi.Nef- viruses (Fig. 2D). Similarly to the difference of GFAP expression between VSVG/GAGi and VSVG/GAGi.Nef- virsues, GFAP protein expression was detected at a higher level in VSVG/NLGi.Nef- -infected U373 than VSVG/NLGi-infceted U373 (~2 fold) (Fig. 2E & F). Next, we performed the similar infection experiments in human primary fetal astrocytes. With a comparable infection effeciency (Fig. 3A), VSVG/NLGi.Nef- infection showed a high level of GFAP protein expression than VSVG/NLGi infection in human primary fetal astrocytes (HPA) (Fig. 3B & C).

### Nef down-regulated GFAP expression at the transcriptional level

**Figure 4.**
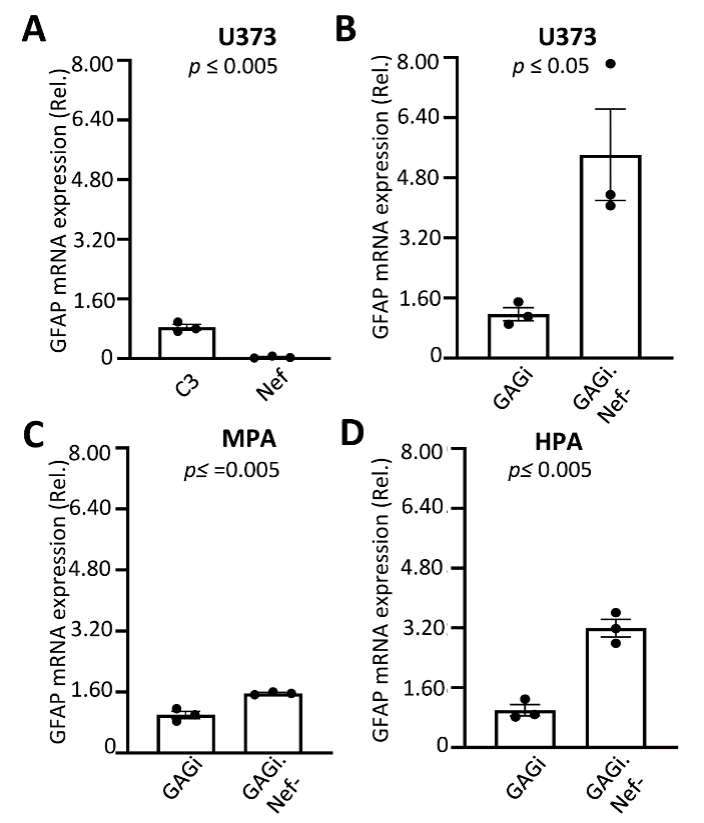
Effects of Nef expression on GFAP mRNA expression in astrocytes. U373 (A & B), mouse primary astrocytes (C), human primary fetal astrocytes (D) were transfected with C3 or Nef (A) or infected with VSVG/GAGi and GAGi.Nef- (B-D). Total RNA was extracted and GFAP mRNA were determined by qRT-PCR. Threshold cycle values were normalized to the mRNA level of GFP which was included as the transfection efficiency control (A) or expressed from VSVG/GAGi and GAGi.Nef- and then normalized to the qRT-PCR control GAPDH and expressed as the relative values to the control C3 (A) or VSVG/GAGi (B-D) for infection efficiency and GAPDH mRNA levels. The data were Mean ± SEM and representative of three independent experiments (n=3).

Regulation of GFAP expresion occurs at both the transcriptional and post-translation levels [20, 22, 58]. To determine if Nef-induced GFAP protein down-modulation resulted from down-modulated transcrption, we first transfected U373 with the Nef expression plasmid, performed qRT-PCR, and determined GFAP mRNA level. Compared to the C3 transfection control, Nef transfection showed ~20 times less GFAP mRNA (Fig. 4A). We also infected U373 with VSVG/GAGi and GAGi.Nef- viruses and determined GFAP mRNA expression. GAGi.Nef- infection showed a higher level of GFAP mRNA than GAGi infection (Fig. 4B). Similar results were obtained in VSVG-pseudotyed virus infected mouse primary astrocytes (Fig. 4C) and human primary fetal astrocytes (Fig. 4D).

### Nef expression inhibited the GFAP promoter activity

**Figure 5.**
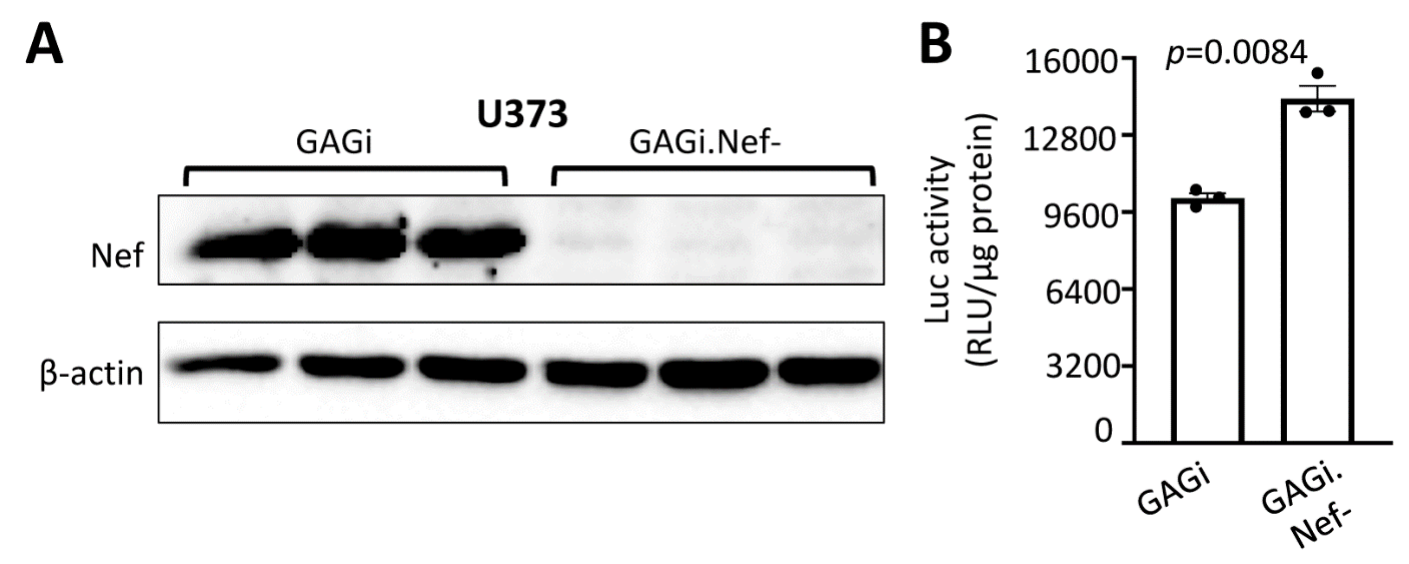
Effects of Nef expression on the GFAP Promoter activity. U373 were transfected with pGFAP.luc plasmid, cultured for 16 hr, split into two equal samples, and infected with VSVG/GAGi and GAGi.Nef-. The cells were cultured for 48 hr and harvested for Western blotting against an anti-Nef, or β-actin antibody (A), or harvested for the luciferase reporter gene assay (B). The relative luciferase activity was normalized to the protein concentration. The data were Mean ± SEM and representative of three independent experiments (n=3).

To further confirm that Nef-induced down-modulation of the GFAP expression occurs at the transcriptional level, we determined effects of HIV infection on the GFAP promoter-driven reporter gene expression. We transfected U373 with the GFAP promoter driven-luciferase reporter gene infected with VSVG/GAGi or GAGi.Nef-, and determined the luciferase gene expression [21, 49, 59]. Expression of Nef protein from VSVG/GAGi infection, confirmed by Westren blotting (Fig. 5A), led to a ~1.4 fold lower level of the luciferase reporter gene activity, compared to that of VSVG/GAGi.Nef- (Fig. 5B). These results were consitent with the changes of GFAP mRNA expression by Nef expression, confirming that Nef-induced down-modulation of GFAP protein expression very likely occurs at the transcriptional level.

### Nef expression altered expression profiles of transcription factors

**Figure 6.**
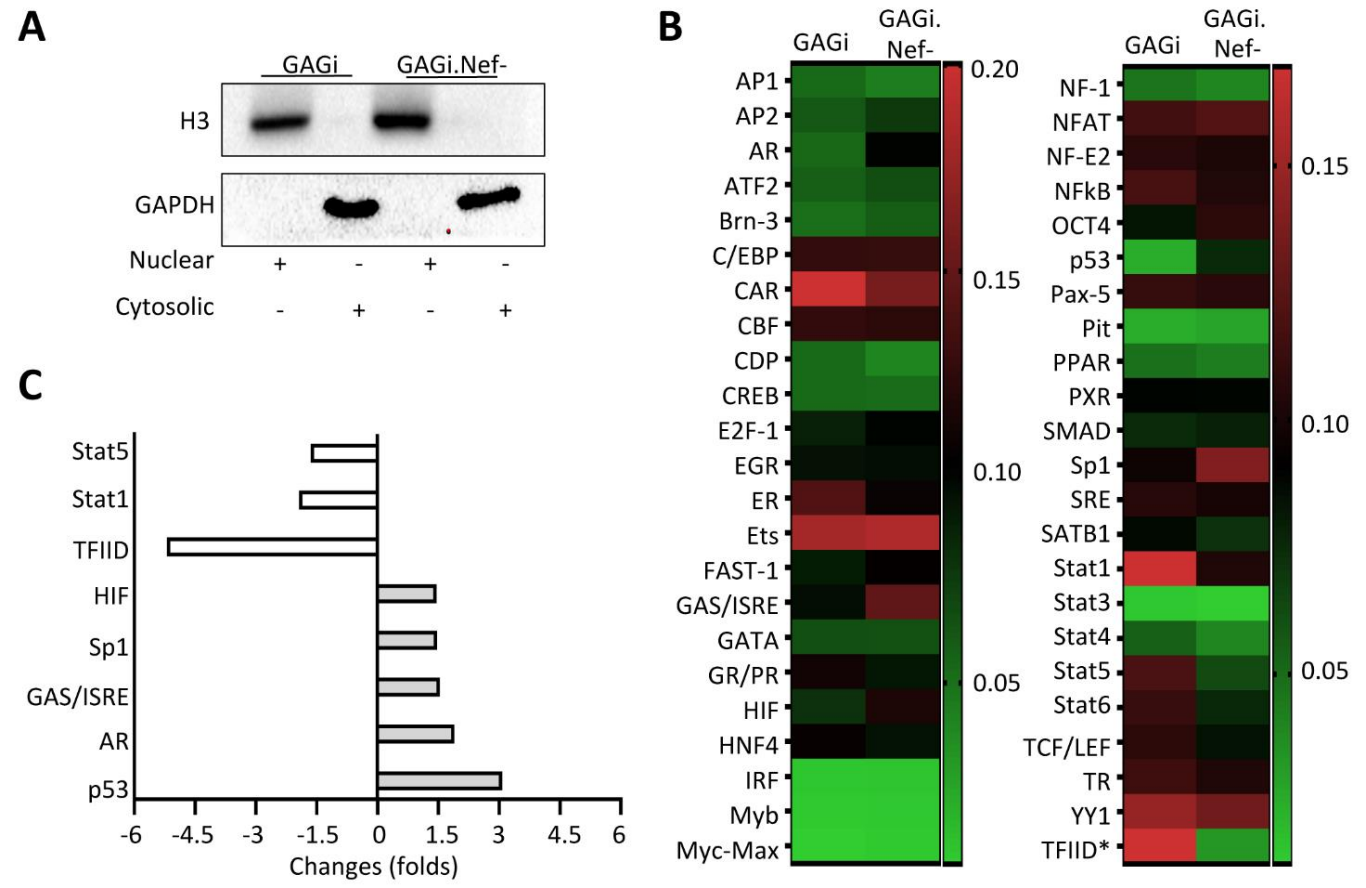
Effects of Nef expression on expression of transcription factors in astrocytes. U373 were infected with VSVG/GAGi and GAGi.Nef-, cultured for 48 hr, and harvested for subcellular fractionations into cytoplasmic and nuclear extracts, as verified by Western blotting against an anti-Histone 3 (H3) antibody as a marker of nuclear extract, or against an anti-GAPDH antibody as a marker of cytoplasmic extract (A), followed by the transcription factor profiling array using the nuclear extracts, differed in expression by the heat map (B) and identification of eight transcription factors with changes of 1.5-fold or higher (three being down-regulated (open bar, C), or five being up-regulated (closed bar, C).

A number of putative DNA binding sites have been identified for various transcription factors within the GFAP promoter [20, 58-61]. Meanwhile, Nef expression has been known to modulate expression of transcription factors and contribute to regulation of host gene expression [55, 60]. To identify possible transcription factors involved in Nef-induced GFAP down-modulation, we performed the transcription factor profiling array. We infected U373 with VSV-G/GAGi or GAGi.Nef- and confirmed a comparable infection efficiency and Nef-induced GFAP down-modulation in VSVG/GAGi-infected cells as before. We then performed subcellular fractionation and prepared cytoplasmic and nuclear extracts of both infected cells. Western blotting against an anti-histone 3 (H3) antibody and anti-GAPDH antibody showed only detection of H3 in the nuclear extract and GAPDH in the cytoplasmic extract (Fig. 6A), suggetsing clear separation of these extracts with undetectable cross contamination. We then used the nuclear extract of both infections for the transcription factor profiling array (Fig. 6B). Among the 46 transcription factors examined were three with 1.5-fold or higher down-regulation (open bar, Fig. 6C) and five with 1.5-fold or higher up-regulation in VSVG/GAGi.Nef- infection compared to VSVG/GAGi infection (closed bar, Fig. 6C). These three down-regulated transcription factors were Stat1, Stat5, and TFIID, while these five up-regulated transcription factors were AR, GAS/ISRE, HIF, Sp1, and p53.

#### Sp1 played an important role in Nef-induced GFAP down-modulation

**Figure 7.**
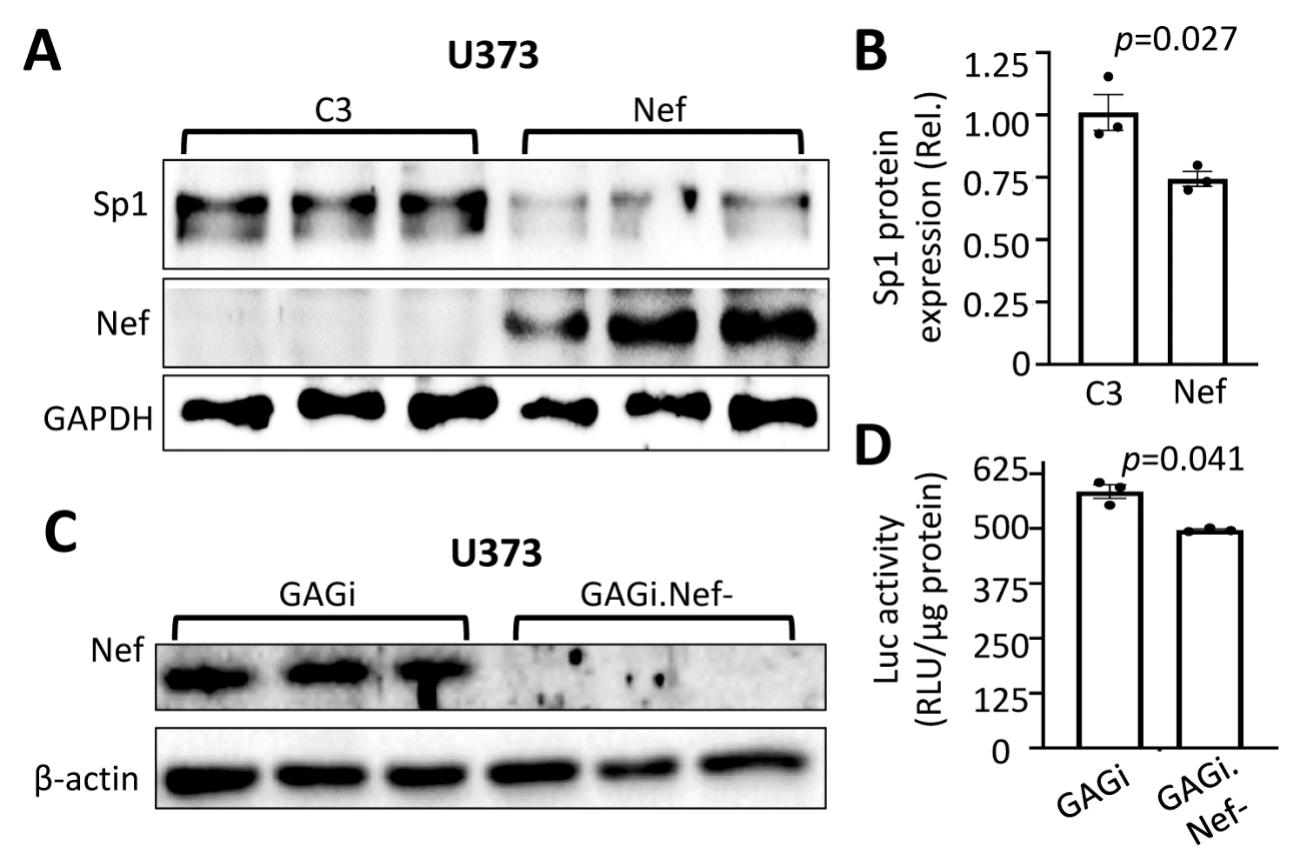
Role of Sp1 in Nef-induced GFAP downmodulation. U373 were transfected with C3 or Nef and harvested for Western blotting against an anti-Sp1, Nef, or GAPDH antibody (A), followed by Sp1 quantitation using the loading control GAPDH as the reference (B); or U373 were transfected with pΔSp1-GFAP.luc plasmid, cultured for 16 hr, split into two equal samples, and infected with VSVG/GAGi and GAGi.Nef-. The cells were cultured for 48 hr and harvested for Western blotting against an anti-Nef, or β-actin antibody (C); or harvested for the luciferase reporter gene assay (D). The relative luciferase activity was normalized to the protein concentration. The data were Mean ± SEM and representative of three independent experiments (n=3).

There are three putative DNA binding sites for transcription factor Sp1 [20, 59, 61]. Knockout of Sp1 leads to decreased GFAP expression and impaired neuron function [62], suggesting that Sp1 is a main positive transcription activator of GFAP gene expression. To determine if Sp1 was involved in Nef-induced GFAP down-modulation, we transfected U373 with Nef and determined Sp1 expression by Western blotting. Compared to the C3 control transfection, Nef expression was associated with a lower level of Sp1 expression (~1.25 times) (Fig. 7A & B). To ascertain the roles of Sp1 in Nef-induced GFAP transcription down-modulation, we deleted these three Sp1 DNA binding sites within the GFAP promoter of the pGFAP.luc reporter gene, transfected U373 with the pΔSp1-GFAP.luc plasmid, infected the cells with VSVG/GAGi or VSV-G/GAGi.Nef-, and determined the luciferase reporter gene expression. Western blotting confirmed Nef expression from VSVG/GAGi infection not VSVG/GAGi.Nef- infection (Fig. 7C). Compared to pGFAP.luc, pΔSp1-GFAP.luc had 23 times lower level of the luciferase expression (Fig. 7D), which was consistent with previous findings[62]. Further, we found that deletion of the Sp1 sites, allowed Nef expression to increase the GFAP promoter activity by 1.2-fold (Fig. 7D). We also determined if Nef expression alone or in the context of HIV infection would alter Sp1 expression and subcellular distribution by immunofluorescence staining, we noticed no changes (Data not shown).

### Nef expression altered glutamate uptake in astrocytes

**Figure 8.**
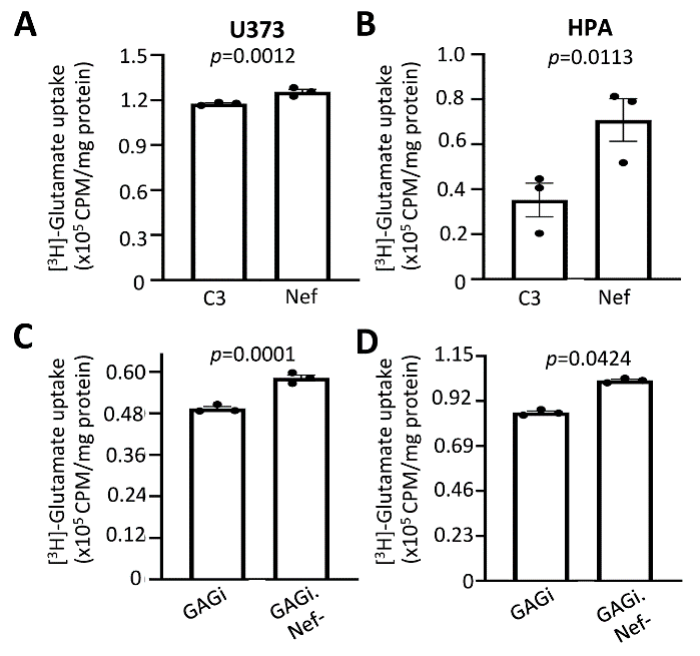
Effects of Nef expression on glutamate uptake into astrocytes. U373 (A & C) or human primary fetal astrocytes (B & D) were transfected with pC3 or Nef (A & B), or infected with VSVG/GAGi and GAGi.Nef- (C & D). The cells were cultured for 24 hr, changed media, pulsed with [^3^H]-glutamate, and harvested for cell lysates. The cell lysates were subjected to scintillation counting and normalized with protein concentration. GFP was included in the transfection to ensure the comparable transfection efficiency (A & B), or GFP expression from the infected cells was used to ensure the comparable infection efficiency (C & D). The data were Mean ± SEM and representative of three independent experiments (n=3).

**Figure 9.**
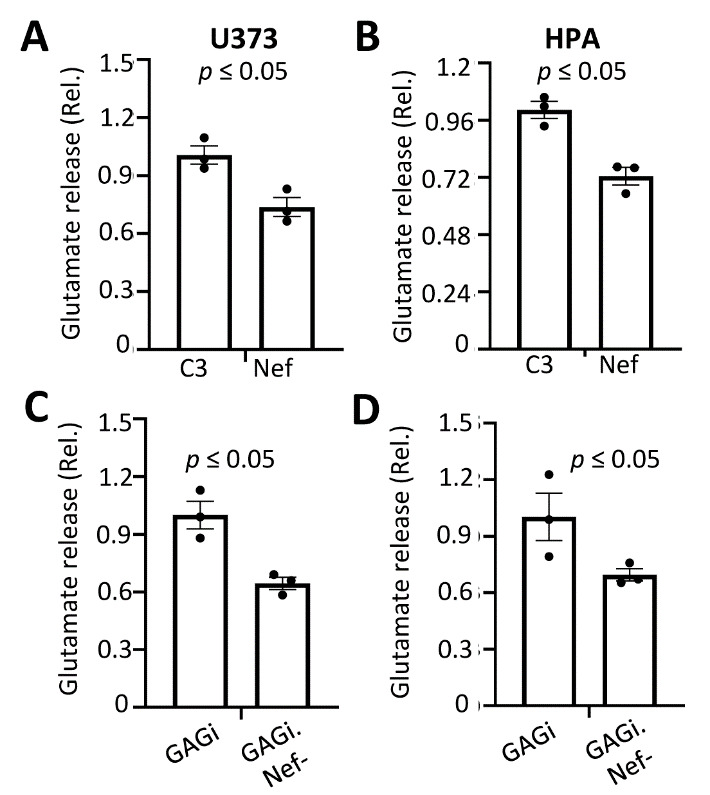
Effects of Nef expression on glutamate release from astrocytes. U373 (A & C) or human primary fetal astrocytes (B & D) were transfected with pC3 or Nef (A & B), or infected with VSVG/GAGi and GAGi.Nef- (C & D). The cells were cultured for 24 hr after changing the media and the glutamate level in the culture media was measured using an absorbance assay and normalized with protein concentration. The data were Mean ± SEM and representative of three independent experiments (n=3).

GFAP is not only a commonly used cellular marker for astrocyte, but its expression plays important roles in astrocyte function [25, 63]. One of the important functions of astrocytes is to maintain homeostasis of neurotransmitters such as glutamate for interactions between astrocytes and neurons, and alterations of the homeostasis of these neurotransmitters could lead to neuropathology [15, 16, 64]. HIV infection has been shown to dysregulate glutamate clearance within the synaptic cleft, resulting in neuroexitotoxicity [65, 66]. Thus, we next determined if Nef-induced GFAP protein down-modulation would alter glutamate metabolism in astrocytes. We transfected U373 and human primary fetal astrocytes with Nef and performed a glutamate uptake assay where cells were pulsed with tritiated glutamate (^3^H-glutamate) and the intracellular glutamate level from the cell lysate was determined using liquid scintillation counting. Nef expression led to ~1.2- and 2-fold higher levels of glutamate in cell lysates than the C3 control in both U373 and primary astrocytes, respectively (Fig. 8A & B). Next, to ascertain the effects of Nef on glutamate uptake during infection, we infected these cells with VSVG-pseudotyped GAGi or GAGi.Nef- and determined the intracellular glutamate levels in the infected cells. To our surprise, Nef expression in the context of GAGi led to a ~1.2 and ~1.35-fold reduction in glutamate uptake levels compared to Nef-null GAGi.Nef- in the infected cells (Fig. 8C & D). As an additional approach for study of glutamate metabolism, glutamate release was also assessed. A colorimetric glutamate assay was employed to determine the levels of glutamate in the culture media. Corresponding to the glutamate uptake results, there was a lower level of glutamate in the supernatant of Nef-transfected cells compared to C3-transfected cells (Fig. 9A & B) and a higher level of glutamate in the culture media from VSVG/GAGi-infected cells than VSVG/GAGi.Nef- -infected cells (Fig. 9C & D). Similar results were obtained both U373 (Fig. 9A & C) and HPA (Fig. 9B & D). Taken together, these data suggest that Nef altered astrocytic regulation of glutamate uptake and release.

### Nef expression enhanced astrocyte proliferation

**Figure 10.**
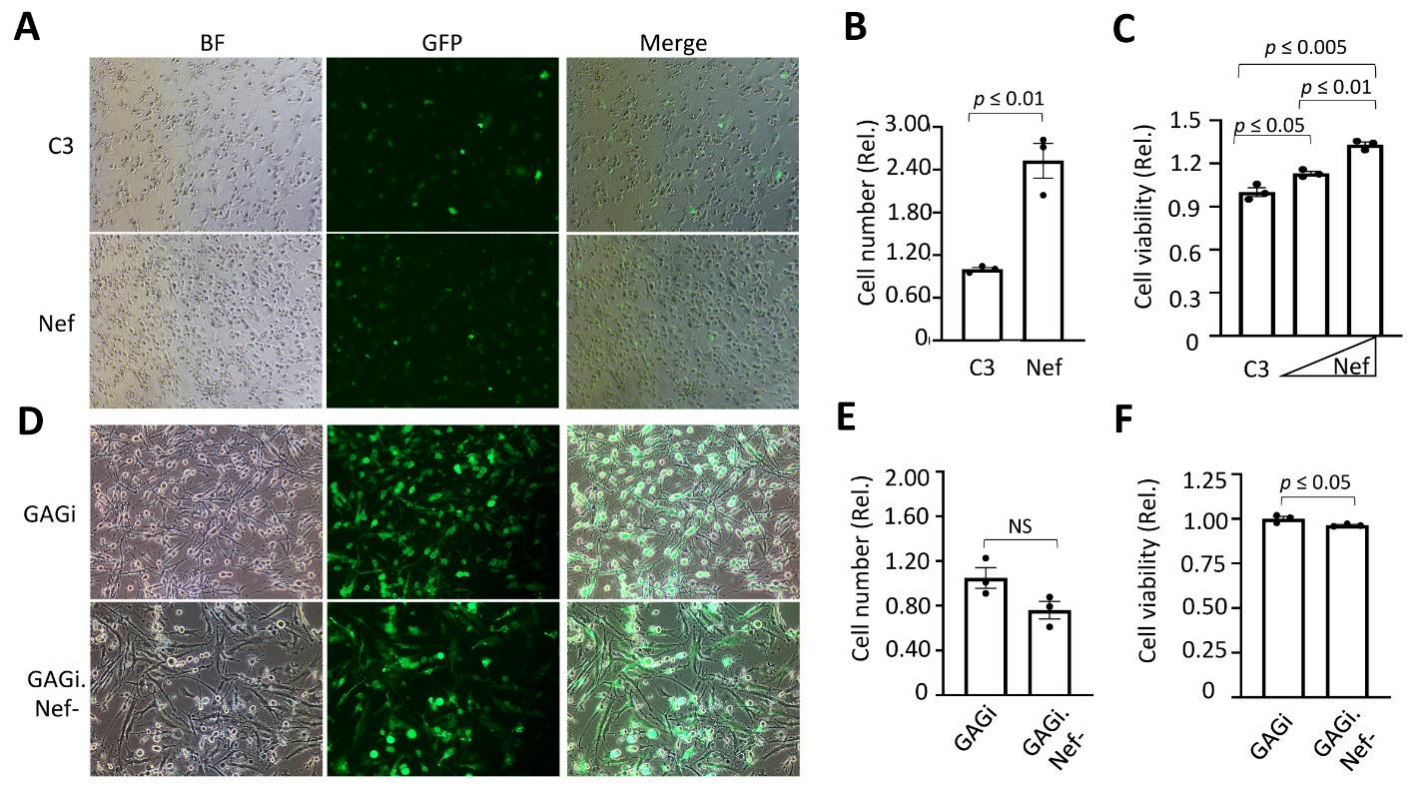
Effects of Nef on astrocyte proliferation. U373 were transfected with pC3 Nef (A, B, & E), or infected with VSVG/GAGi and GAGi.Nef- (C, D & F) and cultured for 48 hr. Brightfield and FITC images were taken at 20x magnification and cells in the brightfield were counted using Image J software (A -D). The MTT assay was performed, and absorbance was measured at 595 nm (background subtraction at 655nm (E &F). The data were Mean ± SEM and representative of three independent experiments (n=3).

We next determined if Nef expression altered astrocyte growth and survival[67]. We transfected U373 with C3 and Nef along with GFP for determination of the transfection efficiency. GFP imaging and quantitation confirmed that the transfection efficiency was comparable (Fig. 10A). Direct counting of cells showed that Nef expression resulted in increases of the cell number (by ~2 fold) compared to cells transfected with C3 (Fig. 10B). The MTT assay also showed the more Nef transfected, the higher the MTT values (Fig. 10C), further showing that Nef promoted astrocyte proliferation. We also infected U373 with GAGi and GAGi.Nef-. The infection efficiency, determined by imaging and counting of GFP+ cells showed a comparable level of infection efficiency (Fig. 10D). GAGi.Nef- showed decreases of the cell number (by ~1.4 fold, albeit the difference did not achieve the significance) compared to GAGi (Fig. 10E). The MTT assay showed significant differences of the cell number between GAGi and Gagi.Nef- -infected cells (by ~1.2 fold) (Fig. 10F). Taken together, these results showed that Nef expression led to enhanced astrocyte proliferation.

## DISCUSSION

NeuroHIV remains highly prevalent among the HIV-infected population, yet its exact cause evades full understanding with no effective therapeutics available. HIV infection of astrocytes and subsequent dysregulation of astrocyte function are known to contribute to the disease [40, 68-70]. With GFAP being a major astrocytic protein involved in maintaining physiological homeostasis of astrocytes, we chose to determine if and how Nef might influence GFAP expression, possibly leading to astrocyte dysfunction. We demonstrated in both human astrocytic cell lines and human primary fetal astrocytes that Nef expression alone or in the context of HIV infection down-modulated GFAP protein expression (Fig. 1-3). We then showed that the down-modulation of GFAP expression occurred at the transcriptional level (Fig. 4-5). Moreover, we showed that transcription factor Sp1 within the GFAP promoter was involved (Fig. 6-7). Finally, we showed that Nef expression impaired glutamate uptake in astrocytes (Fig. 8). Taken together, these data suggest that Nef diminishes GFAP expression through interference of Sp1 expression and activity levels, leading to impaired glutamate metabolism in astrocytes.

GFAP expression and its roles in astrocyte function are not well studied. The protein remains the most infamous astrocytic protein perhaps due to its expression being regarded as a unique marker of astrocyte function used to monitor certain disease states. It is dynamically involved in astrocyte structure and proliferation as a cytoskeletal protein [31, 71]. Additionally, GFAP supports regulation of cellular molecules with mechanosensory properties [21, 72, 73]. Importantly, GFAP aids in regulation of ATP and glutamate levels, maintaining proper neuron function [63, 74]. Knock-out rodent models have provided understanding that GFAP is involved in long-term synaptic potentiation and synaptic depression and is instrumental in astrocyte and brain health during recovery from stress or injury [63, 75, 76]. Fluctuations in GFAP expression outside of homeostatic range propel irregularity in astrocyte functionality. Well-studied irregularities have been documented in diseases such as Alexander’s, Alzheimer’s, epilepsy, and neuroHIV, in which GFAP is mutated or increased during astrocytosis [71, 77-81]. On the other hand, conditions associated with reduced GFAP expression, are attracting more attention. Severe clinical depression, anxiety, and schizophrenia all display diminished GFAP expression [64-66, 68, 69]. Also, reduced GFAP has been linked to certain metabolic conditions such as Diabetes [50, 82]. It is interesting to note that an overall increase in GFAP and resulting manifestation of astrocytosis during neuroHIV, is in large part due to HIV Tat, leading to endoplasmic reticulum stress and lysosomal exocytosis [23, 24].This is in contrast to what we showed in this study that Nef expression resulted in a lower level of GFAP expression (Fig. 1-3) [26, 40, 83]. Conceivably, this may contribute to reports of increased survival of HIV-infected astrocytes, which we noticed during analysis of metabolic activity and microscopic imaging of VSV-G/Gagi infected and Nef transfected U373 cells, when compared to Nef negative counterparts(Fig. 10 A-F) [27, 37, 60, 84, 85] All the findings support the homeostatic threshold hypothesis of GFAP expression and double-edged sword hypothesis of astrocytosis.

Disrupted regulation of GFAP expression is observed in many conditions. But it still remains largely unknown whether and how transcription is involved in the dysregulation. Analysis of the ~2.2 kb GFAP promoter sequence reveals that while the TATA binding factors are not required for activation, there are several DNA binding sites for other major transcription factors such as AP1, NF1, STAT3, NFkB, and Sp1. Interestingly, HIV Tat has been shown to increase GFAP expression through the activation of Egr-1, p300/CREB, and Stat3, yet deletion of Nef did not alter any of these factors, suggesting the mechanism of Nef diminished GFAP expression is unique [86-88].

Our transcription factor profiling of HIV-infected astrocytes did, however, revealed eight transcription factors to be altered by deletion of Nef (Fig. 6). Many of these factors carry out important molecular tasks. Transcription factor II D(TFIID) forms a protein complex, containing the Tata Binding Protein that provides the direct binding sequence targeting the Tata Binding Domain (TBD) of the target mRNA for enhancement of transcription. Several genes are capable of efficient transcription independent of the binding of the TBD. GFAP is among them, hence the activation of TFIID, as observed in our panel with the deletion of Nef, would expectantly not dramatically alter GFAP expression [20, 58, 89]. Viral infection, including HIV infection induce innate and adaptive host immune responses. Key players in this anti-viral response are Type II Interferons, which provide rapid innate defenses early during infection [90-93]. HIV inhibits this host response and literature provides evidence that Nef is involved in blocking IFN reponse [94, 95]. The deletion of Nef leads to activation of Interferon Stimulating Response Elements(GAS/ISRE) transcription factors suggesting that Nef deactivates GAS/ISRE [94-96]. Further, we demonstrated that Signal Transducer and activator of Transcription 1(Stat1) was deactivated with deletion of Nef. IFN-y promotes phosphorylation of Stat1, leading to further enhanced anti-viral responses in astrocytes [92, 94]. Studies have shown that, possibly in a compensatory response due to lack of IFN stimulation, Nef does indeed enhance Stat1 activity, providing additional credence to our profiling panel [97, 98]. Signal Transducer and activator of Transcription 5(Stat5) is an anti-apoptotic factor and while we are among the first to have demonstrated Nef upregulation of Stat5, it is known that Nef inhibits apoptosis [99, 100]. The most well studied mechanism of Nef induced inhibition of apoptosis is via degradation of the host restriction and pro-apoptotic factor p53 [85, 101-104]. Our transcription factor panel coincides with this, demonstrating that the deletion of Nef increased p53 activation (Fig. 6). p53 is delicately regulated by oxygen levels within microenvironments, the HIV-1 *Tat*-interacting protein of 110 kDa (Tip110), and Hypoxia-Inducible factor-1α (HIF-1α) [105]. HIF proteins drive cellular adaption to physiologically low oxygen states, is pivitol during ischemic strokes and brain injury, promoting astrocyte survival, and aids in HIV promoter activation and viral replication [106-108]. The relationship between HIF and p53 is complex with competition for certain co-transactivators such as p300 [109].Because of this, it is reasonable to believe that changes in activation of p53 could also alter HIF activation, which is depicted in our profiling panel with deletion of Nef increasing HIF (Fig. 6). Additionally, deletion of Nef, activated androgen receptor (AR), a ligand-activated receptor for the male steroid hormone Androgen [110, 111]. With the GFAP promoter containing no known binding sites for AR, Androgen/Androgen Receptor complex indirectly and negatively regulates GFAP expression and further, it can be speculated that this regulation is weak due to western blot data displaying decreased GFAP protein in the presence in VSV-G/Gagi infected U373 compared to VSV-G/Gagi.Nef- infected U373 cells (Fig. 6) [112-114].

Of the 8 transcription factors altered by deletion of Nef in this study, Sp1 is unique in having three DNA binding sites within the GFAP promoter and all of these three sites are involved in activation of GFAP expression [20, 59, 61, 115-117]. Sp1 is one of the earliest identified transcription factors within the zinc finger family of proteins and functions as a positive or negative gene transactivator [116, 118]. It regulates ubiquitously expressed and tissue-specific genes and most notably genes involved in cell cycle progression, proliferation, and apoptosis [62, 115, 118]. Its overexpression is linked to tumor and cancer development [119]. It also promotes transcriptional activation of the HIV LTR promoter and increases HIV replication [116]. Knockout of astrocytic Sp1 leads to decreased GFAP expression and compromised neuronal integrity [62], a hallmark of neuroHIV. We identified Sp1 to be among the five down-modulated transcription factors by Nef (Fig. 6), which was confirmed by Western blotting (Fig. 7A & B). Consistent with the findings, we showed that deletion of Sp1 led to a much lower overall level of the GFAP promoter activity (Fig. 7D). To our surprise, we also found that once the Sp1 DNA binding sites were removed from the GFAP promoter, Nef expression exhibited a higher level of the GFAP promoter activity compared to Nef deletion (Fig. 7D). One possible reason for this change is transactivation of other transcription factors within the GFAP promoter AP1, Stat1/5, or NFkB as previously discussed above, by Nef in the absence of Sp1 DNA binding sites [60, 70].

Glutamate is a major excitatory neurotransmitter. NeuroHIV promotes a hyperexcitable and neuroexcitotoxic environment by impairing glutamate transport in neurons, macrophages, and most notably astrocytes [98, 120]. Sodium-dependent excitatory amino acid transportors-1/2 (EAAT-1/2) are predominately expressed in astrocytes and are responsible for rapid bulk removal of glutamate released from neurons[97]. The majority of neuroexcitotory states and subsequent dysfunctions are due to impairments in the EAAT1 and EAAT2 [65, 74, 121]. During neuroHIV, gp120 and Tat are capable of reducing or impairing EAAT2 and glutamate uptake, resulting in neurotoxicity [120, 122, 123]. There has been minimal exploration of Nef induced dysfunction of glutamate clearance. We showed that introduction of Nef into astrocytes led to increased uptake of glutamate (Fig. 8A & B). Extracellular levels of glutamate were also measured revealing a correlating decrease with Nef expression (Fig. 9). Although enhanced uptake is a noncanonical dysfunction, it can lead to loss of neuronal plasticity [63, 97, 124, 125]. Studies show that increased intracellular glutamate alters the gradient and homeostatic balance, leading to astrocyte dysfunction and decreasing EAAT-1/2 sensitivity towards glutamate, ultimately leading to reduced uptake and excitotoxicity [124]. Perhaps a kinetic study of glutamate uptake would provide better understanding. Undoubtably, much remains to be answered on this topic and we attempted to obtain further clarity using a more physiologically relevant model, returning to pseudotyped infection of astrocytes. Interestingly, deletion of Nef during HIV infection, increased glutamate uptake and reduced extracellular glutamate, suggesting that Nef in infected cells negatively impacts glutamate clearance (Fig. 8C & D, Fig 9C & D). Reduction of astrocytic ability to clear the synaptic cleft is known to contribute to HAND and this corresponds to the data from infected cells, but Nef alone increased glutamate uptake in our studies [15, 74]. This was not entirely unexpected. The roles of Nef are variable depending on cell milieu, which is influenced by the location of the expression (peripheral or CNS) and the stage of HIV infection [60, 93, 126]. For instance, Nef is capable of exerting inhibitory or facilitative effects on autophagy dependent on the cell types [60, 127-129]. Additionally, studies show that Nef promotes degradation of p53 leading to inhibition of apoptosis in Nef transfected cells or in HIV infected cells. Contrary to this finding, during infection, Nef induces apoptosis of uninfected bystander cells expressing Nef as well as other viral proteins such as Tat [130, 131]. With this in mind, it can be surmised that Nef might be involved in dynamically compensatory mechanisms in the presence of other viral proteins and has multiple roles in the regulation of glutamate transport in astrocytes.

## Data Availability

All the data reported in this paper will be shared by the corresponding author upon request. Any additional information that may be required to re-analyze the data reported in this paper is available from the corresponding author upon request.
